# The Enhanced Affinity of WRKY Reinforces Drought Tolerance in *Solanum lycopersicum* L.: An Innovative Bioinformatics Study

**DOI:** 10.3390/plants12040762

**Published:** 2023-02-08

**Authors:** Sandip Debnath, Achal Kant, Pradipta Bhowmick, Ayushman Malakar, Shampa Purkaystha, Binod Kumar Jena, Gaurav Mudgal, Mehdi Rahimi, Md Mostofa Uddin Helal, Rakibul Hasan, Jen-Tsung Chen, Faizul Azam

**Affiliations:** 1 Department of Genetics and Plant Breeding, Institute of Agriculture, Visva-Bharati University, Sriniketan 731236, India; 2 Department of Genetics and Plant Breeding, Narayan Institute of Agricultural Sciences, Gopal Narayan Singh University, Sasaram 821305, India; 3 Genetics and Tree Improvement Division, Institute of Forest Productivity (ICFRE), Ranchi 835303, India; 4 Department of Genetics & Plant Breeding and Seed Science & Technology, Centurion University of Technology and Management, Paralakhamundi 761211, India; 5 University Institute of Biotechnology, Chandigarh University, Mohali 140413, India; 6 Department of Biotechnology, Institute of Science and High Technology and Environmental Sciences, Graduate University of Advanced Technology, Kerman 7631885356, Iran; 7 Institute of Wheat Research, State Key Laboratory of Sustainable Dryland Agriculture, Shanxi Agricultural University, Linfen 041000, China; 8 Department of Plant Pathology and Seed Science, Sylhet Agricultural University, Sylhet 3100, Bangladesh; 9 Department of Life Sciences, National University of Kaohsiung, Kaohsiung 811, Taiwan; 10 Department of Pharmaceutical Chemistry and Pharmacognosy, Unaizah College of Pharmacy, Qassim University, Unaizah 51911, Saudi Arabia

**Keywords:** phytochemicals, drought stress, bioinformatics, molecular docking, tomato, curcumin

## Abstract

In the scenario of global climate change, understanding how plants respond to drought is critical for developing future crops that face restricted water resources. This present study focuses on the role of WRKY transcription factors on drought tolerance in tomato, *Solanum lycopersicum* L., which is a significant vegetable crop. WRKY transcription factors are a group of proteins that regulate a wild range of growth and developmental processes in plants such as seed germination and dormancy and the stress response. These transcription factors are defined by the presence of a DNA-binding domain, namely, the WRKY domain. It is well-known that WRKY transcription factors can interact with a variety of proteins and therefore control downstream activities. It aims to simulate the effect of curcumin, a bioactive compound with regulatory capacity, on the protein–protein interaction events by WRKY transcription factors with an emphasis on drought stress. It was found that curcumin binds to WRKY with an energy of −11.43 kcal/mol with inhibitory concentration (K_i_) 0.12 mM and has the potential to improve fruit quality and reinforce drought tolerance of *S. lycopersicum*, according to the results based on bioinformatics tools. The root means square deviation (RMSD) of the C-α, the backbone of 2AYD with ligand coupled complex, displayed a very stable structure with just a little variation of 1.89 Å. MD simulation trajectory of Cα atoms of 2AYD bound to Curcumin revealed more un-ordered orientation in PC1 and PC10 modes and more toward negative correlation from the initial 400 frames during PCA. Establishing the binding energies of the ligand–target interaction is essential in order to characterize the compound’s binding affinity to the drought transcription factor. We think we have identified a phyto-agent called curcumin that has the potential to enhance the drought tolerance. Compared to the part of the mismatch repair-base technique that can be used to fix drought related genes, curcumin performed better in a drop-in crop yield over time, and it was suggested that curcumin is a potential candidate factor for improving drought tolerance in tomatoes, and it needs future validation by experiments in laboratory and field.

## 1. Introduction

**Figure 1:**
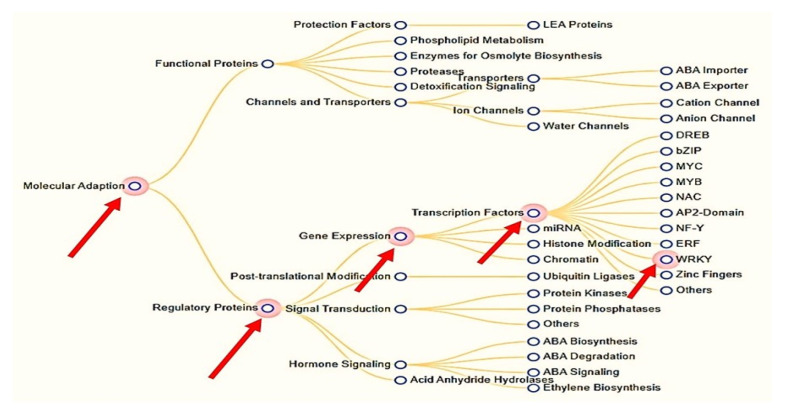
Enhanced lenience to drought and biotic pressure and genes in charge (available from: https://pgsb.helmholtz-muenchen.de/droughtdb/drought_db.html; accessed on: 12 January 2023).

Salt and drought are the main abiotic stress factors for crop plants and all detrimental aspects of climate change that make it difficult to practice sustainable agriculture [1,2,3,4,5,6,7,8]. It is predicted that as greenhouse gas emissions continue, the environment will become much less favorable, limiting agricultural productivity. As a result, there would be an increase in the losses sustained during evaporation and transportation, as well as in the salinity and dryness of the soil and the risk of insect and disease infestation. Besides extreme weather, such as frost or heat, protracted drought or flooding, high soil salt content, or other abiotic situations, are common causes of low yields [1]. Therefore, changes in agro-climatic conditions, particularly the predominance of moisture stress, have a detrimental effect on plant growth, survival, and yield quality [5,6]. Tomatoes are often considered to be one of the most economically important horticultural crops farmed worldwide. In addition, it is an extremely important crop for food production in dry and semi-arid regions. Lack of water is one of the most detrimental abiotic conditions to tomato development. The Mediterranean region is a prime example of this. In addition to a lack of knowledge about the genes that contribute to tomato drought resistance, the processes that govern the plant’s responses to water stress are little understood. Tomatoes may be a commercially significant crop, but this is nonetheless the case. This vegetable is prone to adverse climatic conditions particularly prone to drought [9,10]. Tomatoes cultivated in open fields are more susceptible to damage from abiotic causes than greenhouse-grown crop. These factors include but are not limited to, the length of the growing season, the amount of land available for crop cultivation, and the impacts of drought and extreme heat on crop productivity. When a plant’s roots are chilled below freezing or lower, it loses much of its hygroscopicity. As a result, the water becomes thicker and the crucial membrane found in the roots loses some of its conductivity [10]. As a result, plants may experience water stress in their shoots due to reduced absorption capacity. Tomatoes are unable to lose any more water or produce any more food because their leaves begin to wilt [11]. There is a total of 5936 members of the WRKY transcription factor family that have been found in plants (PlantTFDB 3.0) [6]. Perhaps it can help control how genes are expressed. Their WRKY domain, from which the name is derived, consists of a Cx4-5Cx22-23HxH or Cx7Cx23HxC zinc-finger motif at the end and a generally consistent N-terminal sequence of WRKYGQK [12,13]. Numerous studies have focused on the WRKY domain and its role in gene regulation and cellular communication in plants. There was a common misconception that WRKY transcription factors had a role in plants’ natural defense mechanisms against pathogens [13,14]. Later research has linked them to abiotic stress [14], seed dormancy and germination [15,16], and seed development [17,18]. TTGACC/T W-box sequences in the promoter region are recognized by the vast majority of WRKY proteins [13,19,20], but this does not mean that all WRKY proteins serve the same purpose. Understanding what goes into the W box promoter is not enough to regulate the specificity of WRKY transcription factors. Proteins associated with signal transduction, transcription, and chromatin remodeling may interact with one another in transgenic tobacco, making it more vulnerable to environmental stresses such as cold, curcumin (LN), salt, and dehydration [21]. Ca^2+^-binding signaling molecule calmodulin has been shown to interact with WRKY proteins. Precisely, WRKY have been proven to work together in recent proteomics investigations [21,22,23,24] (as depicted in Figure 1).

During the rapid development phase of drug discovery, natural compounds that are based on the low toxicity profile of small-molecule inhibitors could be very useful. Turmeric’s active ingredient, curcumin, is a polyphenol that is both used medicinally and as a spice for food (*Curcuma longa* Linn). This vibrant yellow spice, which is made from the plant’s rhizome [9], has a long history of use in traditional Chinese and Indian medicine. Since more than 2000 years ago [15], turmeric has been extracted from the root and processed into a powder for use in Asian food, medicine, cosmetics, and fabric coloring. Early explorers brought this essential spice to Europe from Asia for the first time in the 14th century [16]. Curcumin can be a potential compound to inhibit plant fungal attacks and more prone to drought resistance. This research has demonstrated the various ways curcumin can aid in the drought struggle in tomato.

Molecular docking is the method most frequently used in structure-based ligand design because it allows one to consider the action of small molecules in particular protein targets and predict the structure of the ligand–receptor complex [25]. The goal of this study was to search the PubChem database for curcumin, a novel chemical that could be used to improve drought resistance by blocking the WRKY gene’s transcription factor. Researchers used virtual screening and structure-based docking approaches to determine the molecule that inhibits a target the most effectively. Both the compound of interest and the compound that performed the best all-around were examined for structural stability in this study. Studies using molecular dynamics simulations were used in the investigation. To make tomato a viable contender in the battle against drought resistance [2], the main objective of this research is to identify a novel chemical that acts as a potent enhancer of drought resistance in the said vegetable crop.

## 2. Results

### 2.1. Virtual Screening of Compounds

The binding affinity of the complex has a score of −11.43 kcal/mol, and it is denoted as 2AYD-969516. The most promising molecule underwent additional reassembly in the binding cavity of 2AYD. The receptor protein 2AYD, which is shown in Table 1 with an RMSD score of 0.456 angstroms, was subjected to screening with 10 different ligands. This computational analysis of binding energy provided us with a clear picture of the ligand that has the best possible affinity with the protein that was being investigated.

### 2.2. Molecular Docking Investigation

**Figure 2:**
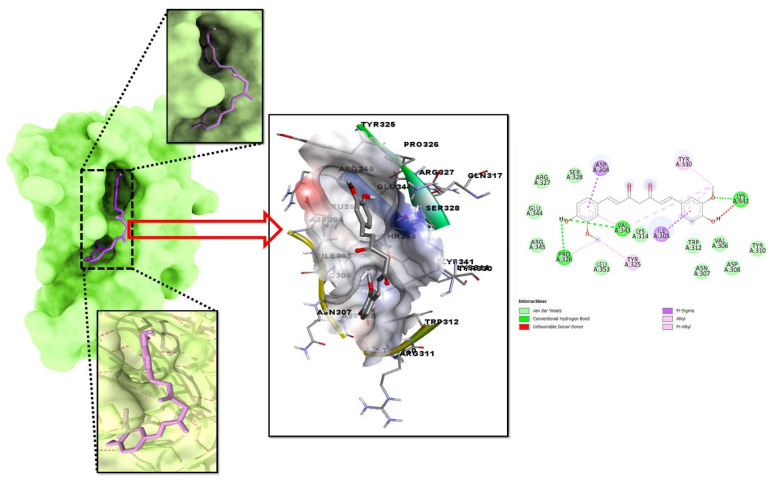
Analysis of the docked posture of 2AYD-969516 displayed the ligand bound at the pocket of the receptor 2AYD and the binding pocket residues interacted with the ligand displayed.

Molecular docking is a method that can be employed to ascertain the intermolecular framework that is optimally shaped by a macromolecule in conjunction with medication or added small molecular contender. At the outset, molecular docking research was conducted to screen for and locate the intermolecular interaction that would be most beneficial between the protein of interest and the phytochemical substances. PyRx instruments: To carry out molecular docking between 10 phytochemical compounds with a three-dimensional structure and desired protein, the AutoDockVina wizard has been used. Table 1 shows the binding affinities of 10 different phytochemicals. Molecular interaction in re-docking studies of ligand Curcumin with 2AYD displayed well-defined binding pockets constituted of residues of leucine, valine, proline, alanine, asparagine, serine, cystine, leucine, and glycine (as depicted in Figure 2) where the ligand bound to the core of the pocket with binding energy (ΔG) −11.43 kcal/mol and inhibitory concentration (K_i_) 0.12 mM.

### 2.3. Molecular Dynamic Simulation Study

**Figure 3:**
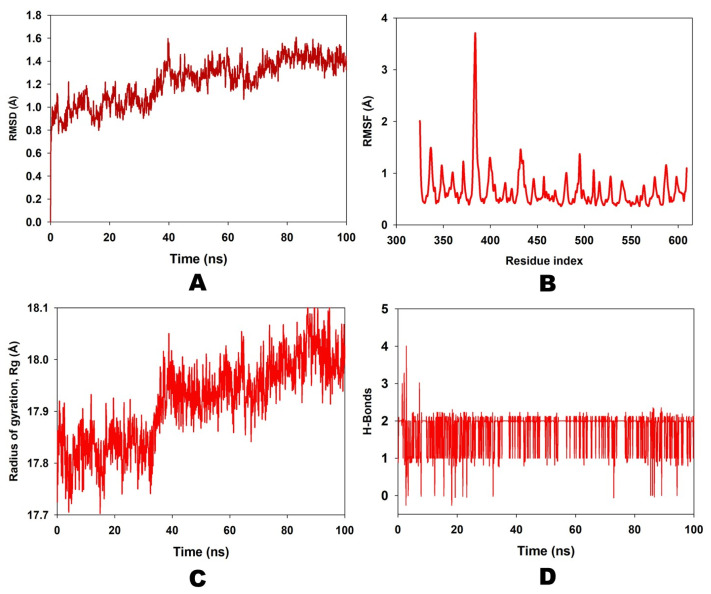
(**A**) RMSD of 2AYD and ligand Curcumin for 100 ns; (**B**) RMSF of 2AYD and ligand Curcumin for 100 ns; (**C**) number of hydrogen bindings of 2AYD and ligand Curcumin for 100 ns; (**D**) radius of gyration of 2AYD and ligand Curcumin for 100 ns.

The ligand Curcumin (PubChem ID: 969516) and the 2AYD protein were subjected to MD simulation studies for one hundred nanoseconds to examine the overall quality and stability of the complex till convergence. The root means square deviation (RMSD) of the C-α, the backbone of 2AYD with ligand coupled complex, revealed an extremely stable structure, with a fluctuation of only 1.89 Å. On the other hand, the RMSD of the ligand Curcumin was slightly distorted in the beginning. However, it remained stable until 100 ns without any significant variations (depicted in Figure 3A). On the other hand, the root means square fluctuations (RMSF) of the different amino acids that make up the C-α backbone of 2AYD showed the most negligible fluctuations, which indicates that the protein structure is stable. The relative mean squared deviation of Curcumin-bound protein simulation trajectories across a timescale of 100 ns. Every piece of data is measured three times to ensure accuracy, and the Y-axis is shifted after each iteration. After a total of 100 ns, the final structure of 2AYD had significant deviations from the reference structure, with an average difference of 2 between it and residue positions 230–250 (depicted in Figure 3B). Figure 3C provides a visual representation of the typical number of hydrogen bonds established between Curcumin and the various proteins throughout the 100 ns simulation. The MD simulation of Curcumin and 2AYD revealed a significant number of hydrogen bonds. Throughout the simulation, a total of two hydrogen bonds were established. The increased number of hydrogen bonds between protein 2AYD and Curcumin has helped to strengthen the binding and enhance the drought resistance, which has contributed to the simulation’s success in maintaining its stability. In addition, the radius of gyration, also known as Rg, was calculated. Rg is an indicator of the size and compactness of the protein when it is in a state where it is attached to the ligand. Figure 3D depicts the Rg plots for convenience. According to the Rg plot of the C-α backbone, the 2AYD protein displays Rg values ranging from 27.8 to 28.0 angstroms, suggesting significant compactness with an average of 0.3 angstroms from the start to the finish of the 100 ns simulation.

**Figure 4:**
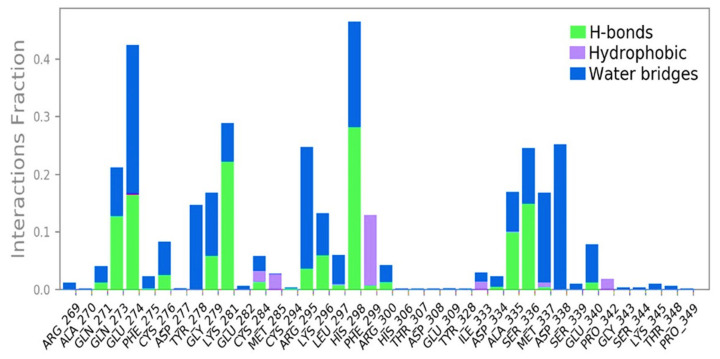
Types of bond formed in 100ns simulation run.

Ligand interaction of Curcumin with predicted docked residues of 2AYD demonstrated the establishment of substantial hydrogen bonds and, apart from this, other non-bonded interactions, such as hydrophobic interaction, as well as water bridges (illustrated in Figure 4). These interactions played a critical role in making a stable complex between the protein and the ligand.

**Figure 5:**
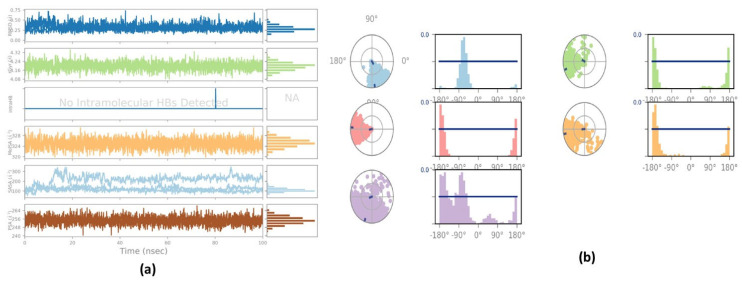
(**a**) The figure displayed shows ligand characteristics such as RMSD, the radius of gyration (rGyr), intra-molecular hydrogen bond, molecular surface area (MolSA), solvent accessible surface area (SASA), and polar surface area (PSA) of Curcumin; (**b**) ligand torsion profile after 100 ns simulation.

The illustration in Figure 5a highlights ligand characteristics such as RMSD, the radius of gyration (rGyr), intramolecular hydrogen bond, molecular surface area (MolSA), solvent accessible surface area (SASA), and polar surface area (PSA). In the ligand, no intramolecular hydrogen was found. In Figure 5b, ligand torsion map shows the structural evolution of each rotatable bond (RB) over time (0.00 through 100.00 ns). Top: Two-dimensional graphic illustrating rotatable ligand linkages. Dial plots and bar plots, both of the same color, indicate rotatable bonds. As the simulation progresses, dial (or radial) charts depict the torsion’s conformation. The simulation is traced in a circle from the display’s center. Dial plots and bar charts depict the torsion’s probability distribution. An infographic shows rotatable bond strength (if torsional potential information is given). Check the chart’s left Y-axis for available values. When conducting this type of research, it is crucial to monitor the histogram, torsion potential, and protein’s conformation strain to evaluate if the bound shape is maintained.

**Figure 6:**
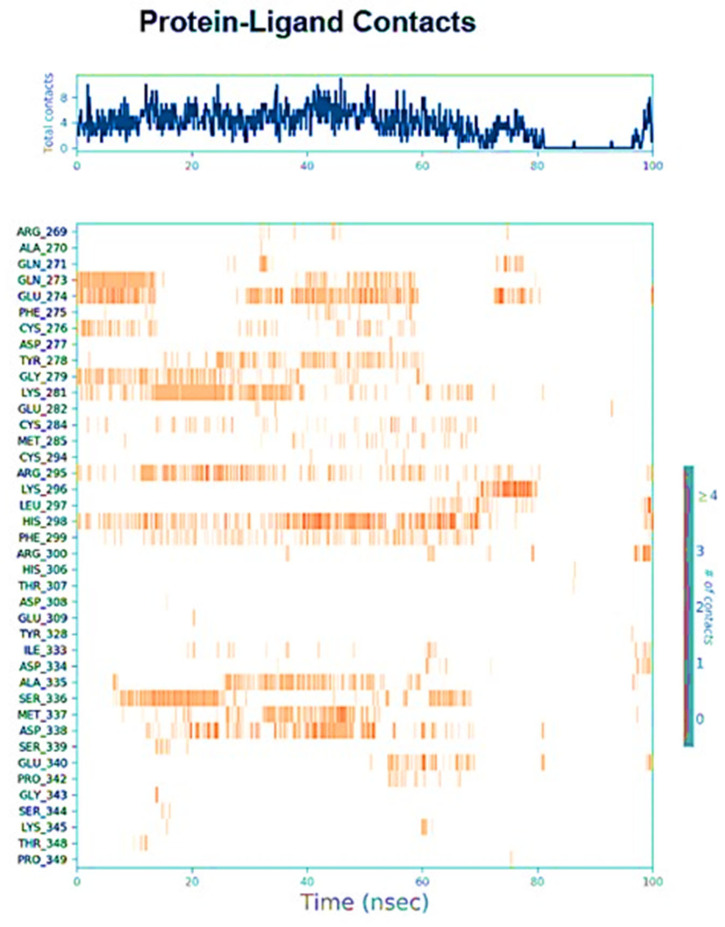
Protein interactions with the ligand can be monitored throughout the simulation.

Protein interactions with the ligand monitored throughout the simulation at 100 ns as depicted in Figure 6.

### 2.4. Molecular Mechanics Generalized Born Surface Area (MM-GBSA) Calculations

Utilizing the MD simulation trajectory, the binding free energy along with other contributing energy in form of MM-GBSA were determined for each 2AYD + Curcumin. The results (Table 2) suggested that the maximum contribution to ΔG_bind_in the stability of the simulated complexes was due to ΔG_bind_Coulomb, ΔG_bind_vdW, and ΔG_bind_Lipo, while, ΔG_bind_Covalent and ΔG_bind_SolvGB contributed to the instability of the corresponding complexes. These results supported the potential of the Curcumin molecule with 2AYD, showed the efficiency in binding to the selected protein, and the ability to form stable protein–ligand complexes.

### 2.5. Principal Component Analysis

**Figure 7:**
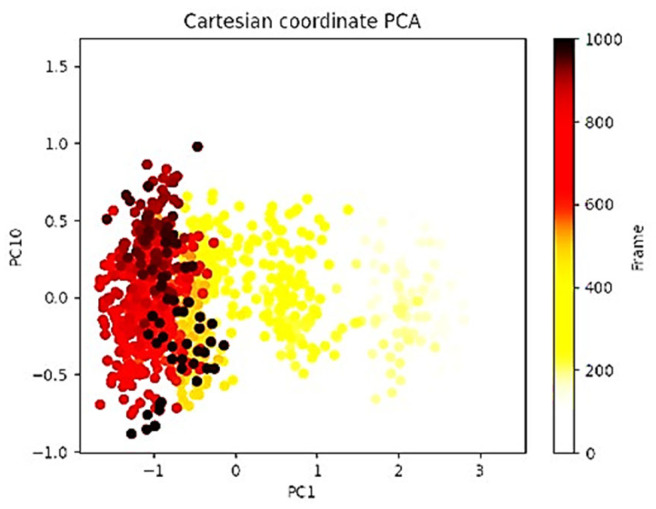
PCA analysis of Eigen values of 1000 frame Cartesian coordinates from MD trajectory for 100 ns of 2AYD + Curcumin.

Principal component analysis (PCA) of the MD simulation trajectories for 2AYD + Curcumin is analyzed to interpret the randomized, global motion of the atoms of amino acid residues and displayed in Figure 7. This analysis interprets the more flexible initial scattered trajectories due to the protein structure’s randomness due to non-correlated global motion. The internal coordinate mobility into three-dimensional space in the spatial time of 100 ns was recorded in a covariance matrix. The rational motion of each trajectory is interpreted in the form of orthogonal sets or Eigenvectors. MD simulation trajectory of Cα atoms of 2AYD bound to Curcumin displayed more unordered orientation in PC1 and PC10 modes and more toward negative correlation from the initial 400 frames (Figure 7) Interestingly, for last 400 frames (from 100 to 400) exhibited positive correlation motion and clustered into more oriented manner. This, therefore, indicated that 2AYD bound to Curcumin centering of the frames in a single cluster (yellow) indicates the periodic motion of MD trajectories due to stable conformational global motion. Therefore, the frames at the end of the simulation become more stable (Figure 7). From the PCA analysis, it can be suggested that all four complexes achieved well stability confronting global correlated motion.

## 3. Materials and Methods

### 3.1. Potential Target Preparation

Accessible from the RCSB protein data bank (PDB) (https://www.rcsb.org/2AYD) are three-dimensional tertiary structures of the β-Secretase protein for study. The structure was imported using a freely available molecular editor (Discovery studio visualizer 4.0). Co-crystal ligands and heteroatoms were deleted before the structure was saved in .pdb format. The Chimera UCSF team employed a thousand-step steepest-descent and a thousand-step Conjugate gradient of energy minimization approach for this optimization. Curcumin (Chem I. D: 92158) was downloaded (https://pubchem.ncbi.nlm.nih.gov/compound/Lupenone; accessed on: 12 January 2023 ) as a .sdf file from PubChem. These .pdb files result from a ligand structure loaded into the Discovery Studio visualizer.

### 3.2. Virtual Screening of Selected Compounds

The active site of an enzyme is a portion of the enzyme that possesses a unique structure that enables it to form a stable bond with a certain type of molecular substrate [26,27]. Due to this, a chemical reaction is triggered in the enzyme. The “active site” of an enzyme is a portion of the enzyme that is shaped in a certain way that allows it to attach to a particular type of molecular substrate. AS helps chemical compounds establish enough contact sites so that they can bind strongly to the enzymes of choice. This allows AS to ensure that the microenvironments for catalysis are adequate and ideal. Therefore, to establish a solid connection between our chemical and the active side of the protein, we examined the active side of the protein using BIOVIA Discovery Studio Visualizer version 19.1.0.18287. This was conducted so that we could achieve our goal. This was in order to create a strong binding affinity in the end product. The binding site that was discovered in the protein complex was also discovered and utilized in the process of creating the receptor grid by making use of the virtual screening application AutoDockVina. Virtual high-throughput screening of ten distinct compounds was carried out with the assistance of AutoDockVina 4.2.6. The compounds were chosen based on the best binding energy scores they achieved with the macromolecule with PDB ID: 2AYD. The best-docked posture with the highest binding energies was chosen for re-docking and additional research out of the nine possible poses for each ligand, which were ranked from top to lowest according to the binding energies they produced.

### 3.3. Molecular Docking Studies

In the AutoDock MGL tool 1.5.6, the receptor protein to be utilized for docking was produced. To construct the receptor grid, residues around 2AYD linked to the co-crystal of Curcumin were utilized. The receptors and ligands were saved in the .pdbqt format using the MGL application so that they could be used in the future. Vina was then launched from a command prompt using the command line. During the configuration process, the grid point spacing was set to its default value of 0.431 angstroms, and the exhaustiveness was set to 8. PyMol and the Discovery Studio Visualizer 2021 were used to examine the output files, which were saved in the .pdbqt format. Using the co-crystallized ligand, the ligand binding was tested for validity and improved upon. The specific molecular mechanism of the target protein is responsible for the binding of Curcumin. The goal of this work is to determine the inhibitory concentration of each candidate molecule and to find the molecule that, according to the results of the virtual screening, is the most effective at interacting with 2AYD. Using the steepest descent method (1000 steps), which was subsequently followed by the addition of the AMBER ff4 force field, the structure of 2AYD was simplified such that it could be more easily understood. This needed to be addressed before the docking study with the essential ligands could begin. Before beginning the investigations into the interaction, the protonation states of the 2AYD that would be involved were tested for neutralization. This was performed before beginning the investigations. With the assistance of AutoDock version 4.2.6, researchers were able to conduct experiments on molecular docking. Polar hydrogen bonds, Kollman and Gastieger charges, and other electrostatic forces were combined to produce not just the receptor, but also the ligands. After merging the nonpolar hydrogens, the receptor and ligand molecules were finally saved in the .pdbqt format. With the values X = 12, Y = 20, and Z = 30, with a spacing of 0.54 angstrom, a grid box was produced. Lamarckian Genetic Algorithm was used to dock protein–ligand complexes to obtain the lowest binding free energy (∆G).

### 3.4. Molecular Dynamic Simulations

Schrodinger, LLC’s Desmond 2020.1 was used to carry out 100 ns MD simulations of the main protein, 2AYD, in conjunction with the ligand Curcumin (C.I.D: 969516). In this particular system, the explicit solvent model with SPC water molecules and the OPLS-2005 force field was utilized [28,29,30]. In order to remove the charge, several Na+ ions were administered. It was decided to add 0.15M NaCl solutions to the system to simulate the physiological environment. The NPT ensemble was generated in each simulation by applying the Nose–Hoover chain coupling method [31,32]. The simulations were run with the following parameters: a temperature of 300 K; a relaxation period of 1.0 ps; a pressure of 1 bar, and a time step of 2 ps after that. The Martyna–Tuckerman–Klein chain coupling system [33] barostat approach was utilized, and the relaxation duration was set at 2 ps. This technique was used to control the pressure. To predict long-range electrostatic interactions, the Colombian interaction radius was fixed at 9, and the particle mesh Ewald technique was utilized [34]. The RESPA integrator was utilized to ascertain the forces that were not bonded. The root mean square deviation was used to evaluate the MD simulations’ capacity to maintain stability (RMSD).

## 4. Discussion

Mismatch repair can be employed as a genetic technique to repair drought genes, but it has been related to a drop-in crop yield in the long run, making curcumin the best option for increasing plants’ tolerance to water stress using competitive inhibition via computational tools. Abiotic stresses (drought, flood, heat, and salt) have a major negative impact on tomato production [1,2], causing yield losses of up to 70% depending on the severity and duration of the stress. Tomatoes are especially vulnerable to the impacts of drought because they lack genes that provide tolerance to this abiotic stress [9,10]. Only 20% of the world’s arable land is irrigated, and only 14% of that is adequately watered. Droughts are typical in such areas, reducing predicted output based on genetic potential and breeding value significantly. Drought largely impacts two aspects of plant–water interactions: ROS generation and tomato plant physiology. While many domesticated and wild plant species have drought tolerance gene(s), employing them is difficult due to the huge genetic distance between them and the obstacles that inhibit embryonic development both before and after transcription. However, computational methods were used to modify the host tomato’s transcriptional genes for drought stress tolerance. The only abiotic element that affects crop production is the intensity of the drought. Plants are fixed and unable to move; therefore, they are more vulnerable to the effects of their environment and humans. If we wish to produce plants that can endure drought, we must first understand how they respond to drought. Proteomic analysis of leaf and root tissues revealed a plethora of drought-responsive proteins, each of which is regulated by a distinct set of genes in the plant genome. Members of the *WRKY* gene family, which is one of the most diverse groups of plant transcription factors, are essential for plant growth and development in response to biotic and abiotic stimuli. The *WRKY* genes are particularly significant because they regulate how plants respond to challenges from both anthropogenic and the natural causes [14,15,16,35,36]. Knowledge of the complicated linkages between WRKY proteins and other cellular components, such as other proteins and ligands, is critical for developing plants that can tolerate biotic and abiotic stressors. This computational study of binding energy supplied us with a clear image of the ligand that had the highest possible affinity for the protein that was being studied. Molecular interaction in re-docking studies of the ligand Curcumin with 2AYD displayed a well-defined binding pocket. This binding pocket was composed of residues including leucine, valine, proline, alanine, asparagine, serine, cystine, leucine, and glycine. The ligand bound to the core of the pocket. These findings provided evidence in favor of the potential of the Curcumin molecule with 2AYD, demonstrated the effectiveness in binding to the chosen protein, and demonstrated the capacity to create stable protein–ligand complexes [25,31,32]. The probability distribution of the torsion may be shown using dial plots and bar charts. The rotatable bond strength is shown in an info-graphic. While carrying out this kind of study, it is essential to keep a close eye on the histogram, the torsion potential, and the conformation strain of the protein in order to determine whether or not the bound shape is retained. Based on the results of the PCA analysis, it is possible to infer that all four complexes have successfully attained stability in the face of global correlated motion.

## 5. Conclusions

We recommend modulating transcription factor activity in response to curcumin, a chemical compound, to improve plant resilience to drought and other difficulties. We conducted molecular dynamics simulations to analyze the predicted structure’s unusual behavior in water. The structure can also be used to assess the complexity of putative ligands or compounds for the WRKY transcription protein. Curcumin’s binding energy to the WRKY protein is −11.43 kcal/mol, indicating a high affinity. Molecular docking and modeling revealed that polyphenols play a crucial role in enhancing drought tolerance in tomato. Understanding the binding energies of the ligand–target interaction is critical for characterizing the compound’s binding affinity to the drought transcription factor-linked target. Finally, we believe we have discovered a phyto-agent called Curcumin that has the potential to improve the drought resistance of cultivated tomato plants.

## Figures and Tables

**Table 1:** Molecular Docking of 10 selected phyto-compounds. Highest binding efficacy was indicated in bold and red.

Sl No.	Compound ID (CID)	Binding Energy (kcal/mol)
1.	CID_100332	−5.6
2.	CID_101389368	−5.5
3.	CID_119034	−6.4
4.	CID_15559069	−7.1
5.	CID_241572	−7.0
**6.**	**CID_ 969516 **	**−11.43**
7.	CID_3981577	−6.9
8.	CID_5280343	−5.3
9.	CID_5280443	−7.0
10.	CID_5280445	−6.2

**Table 2:** Binding free energy components for the 2AYD + Curcumin calculated by MM-GBSA.

Energies (kcal/mol)	Curcumin
ΔG_bind_	−35.56 ± 2.23
ΔG_bind_Lipo	−11.63 ± 1.6
ΔG_bind_vdW	−10.22 ± 2.3
ΔG_bind_Coulomb	−6.22 ± 1.0
ΔG_bind_H_bond_	−0.45 ± 0.2
ΔG_bind_SolvGB	9.36 ± 2.1
ΔG_bind_Covalent	2.75 ± 1.3

## Data Availability

Not applicable.
